# Supplementary material for: Linking citation and retraction data reveals the demographics of scientific retractions among highly cited authors
Source: PLoS Biol. 2025 Jan 30;23(1):e3002999. doi: 10.1371/journal.pbio.3002999 (PMC11781634; doi:10.1371/journal.pbio.3002999)
Supplement: S1 Data — (DOCX) [file pbio.3002999.s001.docx]

S1 Data. Data underlying Fig 2

| Career-long impact | | Single recent year (2023) impact | |
| --- | --- | --- | --- |
| Retractions | Frequency | Retractions | Frequency |
| 1 | 5430 | 1 | 6597 |
| 2 | 957 | 2 | 1199 |
| 3 | 297 | 3 | 360 |
| 4 | 136 | 4 | 172 |
| 5 | 92 | 5 | 116 |
| 6 | 57 | 6 | 69 |
| 7 | 41 | 7 | 53 |
| 8 | 24 | 8 | 36 |
| 9 | 27 | 9 | 32 |
| 10 | 12 | 10 | 16 |
| >10 | 101 | >10 | 97 |
