# Supplementary material for: Linking citation and retraction data reveals the demographics of scientific retractions among highly cited authors
Source: PLoS Biol. 2025 Jan 30;23(1):e3002999. doi: 10.1371/journal.pbio.3002999 (PMC11781634; doi:10.1371/journal.pbio.3002999)
Supplement: S1 Table — (DOCX) [file pbio.3002999.s002.docx]

S1 Table. List of author-attributable reasons used to filter journal error and withdrawn (out of date) exceptions. For a full description of these reasons, please refer to: [https://retractionwatch.com/retraction-watch-database-user-guide/retraction-watch-database-user-guide-appendix-b-reasons/](https://eur03.safelinks.protection.outlook.com/?url=https%3A%2F%2Fretractionwatch.com%2Fretraction-watch-database-user-guide%2Fretraction-watch-database-user-guide-appendix-b-reasons%2F&data=05%7C02%7Cangelo.pezzullo%40unicatt.it%7C8556791578a24faa3e7308dcc8c72da9%7Cb94f7d7481ff44a9b5886682acc85779%7C0%7C0%7C638606005881166835%7CUnknown%7CTWFpbGZsb3d8eyJWIjoiMC4wLjAwMDAiLCJQIjoiV2luMzIiLCJBTiI6Ik1haWwiLCJXVCI6Mn0%3D%7C0%7C%7C%7C&sdata=0TmQUIWd2SN0J2SmPFV0BWztkQCBA1TCie3PAfiJKTo%3D&reserved=0)

| Reason |
| --- |
| Concerns/Issues with Peer Review  Rogue Editor  Unreliable Results  Concerns/Issues about Referencing/Attributions  Concerns/Issues About Data  Concerns/Issues About Results  Plagiarism of Article  Error in Data  Concerns/Issues About Image  Miscommunication by Author  Concerns/Issues About Authorship  Euphemisms for Plagiarism  Fake Peer Review  Error in Analyses  Error in Results and/or Conclusions  Unreliable Data  Not Presented at Conference  Lack of IRB/IACUC Approval  Randomly Generated Content  Hoax Paper  Taken from Dissertation/Thesis  Original Data not Provided  Bias Issues or Lack of Balance  Duplication of Image  Ethical Violations by Author  Conflict of Interest  Plagiarism of Text  Error in Materials (General)  Cites Retracted Work  Breach of Policy by Author |
