# Supplementary material for: Linking citation and retraction data reveals the demographics of scientific retractions among highly cited authors
Source: PLoS Biol. 2025 Jan 30;23(1):e3002999. doi: 10.1371/journal.pbio.3002999 (PMC11781634; doi:10.1371/journal.pbio.3002999)
Supplement: S2 Table — (DOCX) [file pbio.3002999.s003.docx]

S2 Table. Top-cited scientists with and without retracted publications according to their primary subfield

| Primary subfield | Career-long impact | | Single recent year impact | |
| --- | --- | --- | --- | --- |
|  | Retracted | Others | Retracted | Others |
|  | N=7,083 | N=210,014 | N=8,747 | N=214,405 |
| Accounting | 1 (0.8%) | 130 (99.2%) | 2 (1.0%) | 203 (99.0%) |
| Acoustics | 5 (0.7%) | 739 (99.3%) | 9 (1.2%) | 744 (98.8%) |
| Aerospace & Aeronautics | 12 (1.0%) | 1238 (99.0%) | 14 (1.1%) | 1258 (98.9%) |
| Agricultural Economics & Policy | 1 (0.8%) | 127 (99.2%) | 0 (0.0%) | 133 (100.0%) |
| Agronomy & Agriculture | 17 (1.0%) | 1675 (99.0%) | 33 (1.9%) | 1683 (98.1%) |
| Allergy | 13 (3.5%) | 354 (96.5%) | 13 (3.5%) | 363 (96.5%) |
| Analytical Chemistry | 71 (2.7%) | 2511 (97.3%) | 91 (3.5%) | 2515 (96.5%) |
| Anatomy & Morphology | 4 (2.5%) | 156 (97.5%) | 6 (3.6%) | 162 (96.4%) |
| Anesthesiology | 56 (5.3%) | 993 (94.7%) | 47 (4.4%) | 1020 (95.6%) |
| Anthropology | 0 (0.0%) | 193 (100.0%) | 0 (0.0%) | 196 (100.0%) |
| Applied Ethics | 4 (3.1%) | 127 (96.9%) | 3 (2.2%) | 132 (97.8%) |
| Applied Mathematics | 13 (2.8%) | 447 (97.2%) | 34 (7.2%) | 435 (92.8%) |
| Applied Physics | 191 (3.0%) | 6268 (97.0%) | 252 (3.9%) | 6290 (96.1%) |
| Archaeology | 0 (0.0%) | 379 (100.0%) | 1 (0.3%) | 379 (99.7%) |
| Architecture | 0 (0.0%) | 35 (100.0%) | 0 (0.0%) | 39 (100.0%) |
| Art Practice, History & Theory | 0 (0.0%) | 53 (100.0%) | 0 (0.0%) | 54 (100.0%) |
| Arthritis & Rheumatology | 41 (5.0%) | 781 (95.0%) | 47 (5.6%) | 790 (94.4%) |
| Artificial Intelligence & Image Processing | 184 (2.2%) | 8295 (97.8%) | 294 (3.5%) | 8139 (96.5%) |
| Astronomy & Astrophysics | 4 (0.2%) | 1643 (99.8%) | 3 (0.2%) | 1429 (99.8%) |
| Automobile Design & Engineering | 1 (2.3%) | 43 (97.7%) | 2 (4.4%) | 43 (95.6%) |
| Behavioral Science & Comparative Psychology | 9 (2.3%) | 382 (97.7%) | 9 (3.4%) | 255 (96.6%) |
| Biochemistry & Molecular Biology | 313 (7.3%) | 4003 (92.7%) | 354 (8.1%) | 4024 (91.9%) |
| Bioinformatics | 17 (3.7%) | 443 (96.3%) | 14 (3.0%) | 447 (97.0%) |
| Biomedical Engineering | 54 (3.9%) | 1330 (96.1%) | 61 (4.4%) | 1332 (95.6%) |
| Biophysics | 7 (1.7%) | 405 (98.3%) | 12 (2.9%) | 408 (97.1%) |
| Biotechnology | 70 (5.4%) | 1238 (94.6%) | 87 (6.6%) | 1241 (93.4%) |
| Building & Construction | 32 (3.5%) | 871 (96.5%) | 37 (4.0%) | 883 (96.0%) |
| Business & Management | 28 (2.4%) | 1148 (97.6%) | 49 (2.2%) | 2139 (97.8%) |
| Cardiovascular System & Hematology | 284 (6.2%) | 4320 (93.8%) | 279 (6.0%) | 4384 (94.0%) |
| Chemical Engineering | 67 (4.0%) | 1610 (96.0%) | 73 (4.4%) | 1601 (95.6%) |
| Chemical Physics | 46 (2.0%) | 2222 (98.0%) | 64 (2.8%) | 2238 (97.2%) |
| Civil Engineering | 28 (2.3%) | 1209 (97.7%) | 42 (3.4%) | 1210 (96.6%) |
| Classics | 0 (0.0%) | 71 (100.0%) | 0 (0.0%) | 73 (100.0%) |
| Clinical Psychology | 3 (0.8%) | 364 (99.2%) | 5 (1.3%) | 390 (98.7%) |
| Communication & Media Studies | 1 (0.3%) | 288 (99.7%) | 3 (0.7%) | 413 (99.3%) |
| Complementary & Alternative Medicine | 23 (7.9%) | 268 (92.1%) | 31 (10.5%) | 263 (89.5%) |
| Computation Theory & Mathematics | 0 (0.0%) | 422 (100.0%) | 1 (0.2%) | 425 (99.8%) |
| Computer Hardware & Architecture | 0 (0.0%) | 403 (100.0%) | 1 (0.2%) | 410 (99.8%) |
| Criminology | 6 (2.2%) | 261 (97.8%) | 6 (2.2%) | 267 (97.8%) |
| Cultural Studies | 0 (0.0%) | 170 (100.0%) | 0 (0.0%) | 170 (100.0%) |
| Dairy & Animal Science | 19 (1.3%) | 1453 (98.7%) | 35 (2.3%) | 1463 (97.7%) |
| Demography | 0 (0.0%) | 76 (100.0%) | 0 (0.0%) | 79 (100.0%) |
| Dentistry | 43 (2.3%) | 1816 (97.7%) | 72 (3.8%) | 1813 (96.2%) |
| Dermatology & Venereal Diseases | 42 (3.5%) | 1143 (96.5%) | 56 (4.6%) | 1164 (95.4%) |
| Design Practice & Management | 4 (1.7%) | 231 (98.3%) | 7 (2.9%) | 233 (97.1%) |
| Development Studies | 1 (1.0%) | 99 (99.0%) | 0 (0.0%) | 106 (100.0%) |
| Developmental & Child Psychology | 9 (1.4%) | 617 (98.6%) | 11 (1.6%) | 675 (98.4%) |
| Developmental Biology | 240 (7.8%) | 2824 (92.2%) | 206 (6.7%) | 2878 (93.3%) |
| Distributed Computing | 1 (0.5%) | 202 (99.5%) | 2 (1.0%) | 207 (99.0%) |
| Drama & Theater | 0 (0.0%) | 25 (100.0%) | 0 (0.0%) | 27 (100.0%) |
| Ecology | 32 (1.9%) | 1682 (98.1%) | 36 (1.9%) | 1872 (98.1%) |
| Econometrics | 0 (0.0%) | 75 (100.0%) | 0 (0.0%) | 86 (100.0%) |
| Economic Theory | 0 (0.0%) | 35 (100.0%) | 0 (0.0%) | 36 (100.0%) |
| Economics | 6 (0.6%) | 970 (99.4%) | 14 (1.1%) | 1241 (98.9%) |
| Education | 8 (0.4%) | 1992 (99.6%) | 14 (0.7%) | 2015 (99.3%) |
| Electrical & Electronic Engineering | 47 (1.9%) | 2482 (98.1%) | 59 (2.3%) | 2497 (97.7%) |
| Emergency & Critical Care Medicine | 16 (2.0%) | 799 (98.0%) | 18 (2.2%) | 803 (97.8%) |
| Endocrinology & Metabolism | 155 (7.3%) | 1961 (92.7%) | 136 (7.1%) | 1767 (92.9%) |
| Energy | 230 (3.5%) | 6389 (96.5%) | 310 (4.6%) | 6362 (95.4%) |
| Entomology | 8 (1.0%) | 758 (99.0%) | 13 (1.7%) | 761 (98.3%) |
| Environmental & Occupational Health | 8 (2.6%) | 299 (97.4%) | 8 (2.6%) | 299 (97.4%) |
| Environmental Engineering | 27 (2.1%) | 1235 (97.9%) | 42 (3.3%) | 1236 (96.7%) |
| Environmental Sciences | 113 (4.7%) | 2286 (95.3%) | 169 (7.0%) | 2253 (93.0%) |
| Epidemiology | 8 (4.0%) | 191 (96.0%) | 7 (3.5%) | 194 (96.5%) |
| Evolutionary Biology | 26 (3.1%) | 813 (96.9%) | 21 (3.3%) | 616 (96.7%) |
| Experimental Psychology | 30 (2.5%) | 1193 (97.5%) | 32 (2.6%) | 1181 (97.4%) |
| Family Studies | 0 (0.0%) | 78 (100.0%) | 0 (0.0%) | 76 (100.0%) |
| Finance | 3 (1.1%) | 280 (98.9%) | 5 (1.3%) | 389 (98.7%) |
| Fisheries | 19 (2.2%) | 835 (97.8%) | 21 (2.4%) | 860 (97.6%) |
| Fluids & Plasmas | 14 (1.2%) | 1107 (98.8%) | 12 (1.1%) | 1129 (98.9%) |
| Folklore | 0 (0.0%) | 14 (100.0%) | 0 (0.0%) | 15 (100.0%) |
| Food Science | 43 (2.5%) | 1701 (97.5%) | 73 (4.1%) | 1701 (95.9%) |
| Forestry | 3 (0.4%) | 689 (99.6%) | 10 (1.4%) | 698 (98.6%) |
| Gastroenterology & Hepatology | 123 (5.8%) | 1986 (94.2%) | 128 (5.9%) | 2024 (94.1%) |
| Gender Studies | 0 (0.0%) | 48 (100.0%) | 0 (0.0%) | 56 (100.0%) |
| General & Internal Medicine | 148 (2.1%) | 6741 (97.9%) | 201 (2.9%) | 6721 (97.1%) |
| General Chemistry | 29 (2.6%) | 1089 (97.4%) | 37 (3.3%) | 1100 (96.7%) |
| General Clinical Medicine | 12 (2.5%) | 459 (97.5%) | 12 (2.5%) | 472 (97.5%) |
| General Mathematics | 27 (1.8%) | 1464 (98.2%) | 39 (2.6%) | 1471 (97.4%) |
| General Physics | 28 (1.7%) | 1657 (98.3%) | 36 (2.2%) | 1633 (97.8%) |
| General Psychology & Cognitive Sciences | 3 (3.8%) | 77 (96.2%) | 4 (5.0%) | 76 (95.0%) |
| Genetics & Heredity | 29 (3.6%) | 784 (96.4%) | 27 (3.3%) | 793 (96.7%) |
| Geochemistry & Geophysics | 24 (1.2%) | 2028 (98.8%) | 33 (1.6%) | 2038 (98.4%) |
| Geography | 4 (1.0%) | 405 (99.0%) | 6 (1.1%) | 523 (98.9%) |
| Geological & Geomatics Engineering | 25 (1.7%) | 1423 (98.3%) | 43 (2.9%) | 1415 (97.1%) |
| Geology | 2 (0.6%) | 352 (99.4%) | 3 (0.8%) | 359 (99.2%) |
| Geriatrics | 10 (3.8%) | 250 (96.2%) | 13 (4.9%) | 250 (95.1%) |
| Gerontology | 3 (1.2%) | 240 (98.8%) | 1 (0.4%) | 237 (99.6%) |
| Health Policy & Services | 7 (1.6%) | 439 (98.4%) | 8 (1.8%) | 445 (98.2%) |
| History | 0 (0.0%) | 363 (100.0%) | 1 (0.3%) | 355 (99.7%) |
| History of Science, Technology & Medicine | 0 (0.0%) | 65 (100.0%) | 0 (0.0%) | 66 (100.0%) |
| History of Social Sciences | 0 (0.0%) | 47 (100.0%) | 0 (0.0%) | 47 (100.0%) |
| Horticulture | 0 (0.0%) | 114 (100.0%) | 0 (0.0%) | 117 (100.0%) |
| Human Factors | 2 (0.6%) | 308 (99.4%) | 2 (0.6%) | 311 (99.4%) |
| Immunology | 289 (9.0%) | 2940 (91.0%) | 270 (8.2%) | 3003 (91.8%) |
| Industrial Engineering & Automation | 58 (2.4%) | 2337 (97.6%) | 70 (2.9%) | 2334 (97.1%) |
| Industrial Relations | 1 (2.0%) | 48 (98.0%) | 1 (2.0%) | 49 (98.0%) |
| Information & Library Sciences | 1 (0.3%) | 302 (99.7%) | 2 (0.6%) | 314 (99.4%) |
| Information Systems | 2 (0.5%) | 382 (99.5%) | 5 (1.3%) | 375 (98.7%) |
| Inorganic & Nuclear Chemistry | 36 (2.2%) | 1581 (97.8%) | 55 (3.3%) | 1590 (96.7%) |
| International Relations | 0 (0.0%) | 177 (100.0%) | 0 (0.0%) | 179 (100.0%) |
| Languages & Linguistics | 1 (0.2%) | 444 (99.8%) | 1 (0.2%) | 446 (99.8%) |
| Law | 0 (0.0%) | 293 (100.0%) | 0 (0.0%) | 302 (100.0%) |
| Legal & Forensic Medicine | 13 (4.3%) | 291 (95.7%) | 17 (5.6%) | 286 (94.4%) |
| Literary Studies | 0 (0.0%) | 377 (100.0%) | 0 (0.0%) | 377 (100.0%) |
| Logistics & Transportation | 11 (1.8%) | 595 (98.2%) | 10 (1.6%) | 598 (98.4%) |
| Marine Biology & Hydrobiology | 7 (0.7%) | 1042 (99.3%) | 10 (0.9%) | 1056 (99.1%) |
| Marketing | 6 (2.1%) | 284 (97.9%) | 15 (2.5%) | 575 (97.5%) |
| Materials | 221 (3.5%) | 6086 (96.5%) | 294 (4.6%) | 6089 (95.4%) |
| Mathematical Physics | 3 (2.2%) | 132 (97.8%) | 4 (2.9%) | 136 (97.1%) |
| Mechanical Engineering & Transports | 121 (3.9%) | 2944 (96.1%) | 183 (6.0%) | 2888 (94.0%) |
| Medical Informatics | 5 (1.3%) | 371 (98.7%) | 5 (1.3%) | 372 (98.7%) |
| Medicinal & Biomolecular Chemistry | 125 (5.6%) | 2111 (94.4%) | 161 (7.1%) | 2107 (92.9%) |
| Meteorology & Atmospheric Sciences | 26 (1.6%) | 1644 (98.4%) | 35 (1.8%) | 1888 (98.2%) |
| Microbiology | 152 (3.6%) | 4076 (96.4%) | 168 (3.9%) | 4097 (96.1%) |
| Microscopy | 3 (3.6%) | 80 (96.4%) | 4 (4.8%) | 79 (95.2%) |
| Mining & Metallurgy | 13 (1.8%) | 725 (98.2%) | 18 (2.4%) | 726 (97.6%) |
| Music | 0 (0.0%) | 73 (100.0%) | 0 (0.0%) | 76 (100.0%) |
| Mycology & Parasitology | 13 (2.3%) | 560 (97.7%) | 15 (2.6%) | 572 (97.4%) |
| Nanoscience & Nanotechnology | 121 (4.3%) | 2698 (95.7%) | 155 (4.1%) | 3596 (95.9%) |
| Networking & Telecommunications | 90 (2.1%) | 4280 (97.9%) | 158 (3.6%) | 4246 (96.4%) |
| Neurology & Neurosurgery | 305 (4.3%) | 6753 (95.7%) | 354 (5.0%) | 6792 (95.0%) |
| Nuclear & Particle Physics | 25 (0.8%) | 3209 (99.2%) | 27 (0.8%) | 3283 (99.2%) |
| Nuclear Medicine & Medical Imaging | 54 (2.2%) | 2363 (97.8%) | 62 (2.5%) | 2409 (97.5%) |
| Numerical & Computational Mathematics | 3 (0.9%) | 348 (99.1%) | 7 (2.0%) | 349 (98.0%) |
| Nursing | 27 (2.2%) | 1228 (97.8%) | 35 (2.7%) | 1249 (97.3%) |
| Nutrition & Dietetics | 26 (2.5%) | 1035 (97.5%) | 36 (3.3%) | 1045 (96.7%) |
| Obstetrics & Reproductive Medicine | 82 (4.1%) | 1910 (95.9%) | 86 (4.3%) | 1929 (95.7%) |
| Oceanography | 3 (0.9%) | 335 (99.1%) | 2 (0.6%) | 336 (99.4%) |
| Oncology & Carcinogenesis | 575 (8.4%) | 6287 (91.6%) | 687 (9.9%) | 6263 (90.1%) |
| Operations Research | 10 (1.6%) | 615 (98.4%) | 14 (2.3%) | 606 (97.7%) |
| Ophthalmology & Optometry | 66 (4.1%) | 1527 (95.9%) | 76 (4.7%) | 1534 (95.3%) |
| Optics | 18 (1.1%) | 1642 (98.9%) | 20 (1.2%) | 1658 (98.8%) |
| Optoelectronics & Photonics | 37 (1.4%) | 2574 (98.6%) | 52 (1.9%) | 2616 (98.1%) |
| Organic Chemistry | 102 (2.9%) | 3466 (97.1%) | 151 (4.2%) | 3469 (95.8%) |
| Ornithology | 2 (1.6%) | 125 (98.4%) | 3 (2.3%) | 129 (97.7%) |
| Orthopedics | 50 (2.9%) | 1667 (97.1%) | 71 (4.1%) | 1667 (95.9%) |
| Otorhinolaryngology | 20 (1.9%) | 1006 (98.1%) | 22 (2.1%) | 1029 (97.9%) |
| Paleontology | 7 (1.4%) | 496 (98.6%) | 7 (1.4%) | 476 (98.6%) |
| Pathology | 21 (4.2%) | 481 (95.8%) | 21 (4.1%) | 491 (95.9%) |
| Pediatrics | 34 (2.2%) | 1485 (97.8%) | 45 (2.9%) | 1504 (97.1%) |
| Pharmacology & Pharmacy | 162 (5.2%) | 2935 (94.8%) | 293 (9.4%) | 2838 (90.6%) |
| Philosophy | 0 (0.0%) | 262 (100.0%) | 0 (0.0%) | 268 (100.0%) |
| Physical Chemistry | 19 (2.2%) | 830 (97.8%) | 23 (2.7%) | 821 (97.3%) |
| Physiology | 13 (2.5%) | 517 (97.5%) | 17 (3.2%) | 519 (96.8%) |
| Plant Biology & Botany | 165 (4.6%) | 3459 (95.4%) | 237 (6.4%) | 3441 (93.6%) |
| Political Science & Public Administration | 1 (0.2%) | 494 (99.8%) | 1 (0.1%) | 806 (99.9%) |
| Polymers | 63 (2.6%) | 2391 (97.4%) | 86 (3.5%) | 2387 (96.5%) |
| Psychiatry | 81 (4.5%) | 1712 (95.5%) | 78 (4.6%) | 1604 (95.4%) |
| Psychoanalysis | 0 (0.0%) | 74 (100.0%) | 0 (0.0%) | 78 (100.0%) |
| Public Health | 33 (2.3%) | 1409 (97.7%) | 29 (2.0%) | 1429 (98.0%) |
| Rehabilitation | 5 (0.8%) | 591 (99.2%) | 6 (1.0%) | 604 (99.0%) |
| Religions & Theology | 0 (0.0%) | 218 (100.0%) | 1 (0.4%) | 224 (99.6%) |
| Respiratory System | 77 (5.7%) | 1274 (94.3%) | 76 (5.5%) | 1298 (94.5%) |
| Science Studies | 3 (3.0%) | 97 (97.0%) | 4 (2.9%) | 134 (97.1%) |
| Social Psychology | 38 (4.7%) | 779 (95.3%) | 45 (4.1%) | 1046 (95.9%) |
| Social Sciences Methods | 0 (0.0%) | 110 (100.0%) | 0 (0.0%) | 137 (100.0%) |
| Social Work | 0 (0.0%) | 183 (100.0%) | 0 (0.0%) | 179 (100.0%) |
| Sociology | 0 (0.0%) | 271 (100.0%) | 1 (0.2%) | 461 (99.8%) |
| Software Engineering | 4 (0.8%) | 470 (99.2%) | 4 (0.8%) | 471 (99.2%) |
| Speech-Language Pathology & Audiology | 4 (1.6%) | 247 (98.4%) | 4 (1.6%) | 254 (98.4%) |
| Sport Sciences | 18 (2.5%) | 696 (97.5%) | 26 (3.6%) | 696 (96.4%) |
| Sport, Leisure & Tourism | 3 (1.5%) | 196 (98.5%) | 9 (2.0%) | 432 (98.0%) |
| Statistics & Probability | 8 (1.7%) | 458 (98.3%) | 10 (2.0%) | 480 (98.0%) |
| Strategic, Defence & Security Studies | 3 (0.6%) | 520 (99.4%) | 4 (0.8%) | 521 (99.2%) |
| Substance Abuse | 4 (1.1%) | 355 (98.9%) | 5 (1.4%) | 343 (98.6%) |
| Surgery | 94 (3.4%) | 2639 (96.6%) | 101 (3.6%) | 2704 (96.4%) |
| Toxicology | 56 (4.1%) | 1296 (95.9%) | 63 (4.6%) | 1311 (95.4%) |
| Tropical Medicine | 16 (2.1%) | 754 (97.9%) | 17 (2.2%) | 771 (97.8%) |
| Urban & Regional Planning | 2 (0.7%) | 265 (99.3%) | 2 (0.7%) | 304 (99.3%) |
| Urology & Nephrology | 95 (5.3%) | 1696 (94.7%) | 102 (5.6%) | 1708 (94.4%) |
| Veterinary Sciences | 16 (1.2%) | 1296 (98.8%) | 27 (2.0%) | 1304 (98.0%) |
| Virology | 78 (5.3%) | 1403 (94.7%) | 78 (5.2%) | 1423 (94.8%) |
| Zoology | 0 (0.0%) | 388 (100.0%) | 2 (0.5%) | 398 (99.5%) |
