# Supplementary material for: Linking citation and retraction data reveals the demographics of scientific retractions among highly cited authors
Source: PLoS Biol. 2025 Jan 30;23(1):e3002999. doi: 10.1371/journal.pbio.3002999 (PMC11781634; doi:10.1371/journal.pbio.3002999)
Supplement: S3 Table — (DOCX) [file pbio.3002999.s004.docx]

S3 Table. Top-cited scientists with and without retracted publications in countries with high (>10%) retraction prevalence

| Country | Career-long impact | | Single recent year impact | |
| --- | --- | --- | --- | --- |
|  | Retracted | Others | Retracted | Others |
| Armenia | 0 (0.0%) | 7 (100.0%) | 1 (11.1%) | 8 (88.9%) |
| Azerbaijan | 1 (11.1%) | 8 (88.9%) | 2 (11.1%) | 16 (88.9%) |
| Bangladesh | 8 (11.8%) | 60 (88.2%) | 19 (9.3%) | 186 (90.7%) |
| Belarus | 2 (8.7%) | 21 (91.3%) | 4 (26.7%) | 11 (73.3%) |
| Ecuador | 6 (28.6%) | 15 (71.4%) | 7 (28.0%) | 18 (72.0%) |
| Grenada | 1 (16.7%) | 5 (83.3%) | 1 (25.0%) | 3 (75.0%) |
| Iran | 128 (12.5%) | 892 (87.5%) | 245 (10.5%) | 2082 (89.5%) |
| Iraq | 7 (14.3%) | 42 (85.7%) | 33 (15.8%) | 176 (84.2%) |
| Jamaica | 1 (6.7%) | 14 (93.3%) | 1 (11.1%) | 8 (88.9%) |
| Kazakhstan | 5 (17.2%) | 24 (82.8%) | 6 (14.6%) | 35 (85.4%) |
| Kyrgyzstan | 0 (0.0%) | 2 (100.0%) | 1 (50.0%) | 1 (50.0%) |
| Kuwait | 4 (5.5%) | 69 (94.5%) | 10 (14.5%) | 59 (85.5%) |
| Liberia | 1 (25.0%) | 3 (75.0%) | 1 (10.0%) | 9 (90.0%) |
| Montenegro | 0 (0.0%) | 5 (100.0%) | 1 (20.0%) | 4 (80.0%) |
| Mauritius | 1 (16.7%) | 5 (83.3%) | 1 (10.0%) | 9 (90.0%) |
| Malaysia | 37 (10.1%) | 331 (89.9%) | 69 (9.0%) | 700 (91.0%) |
| Nepal | 1 (12.5%) | 7 (87.5%) | 1 (3.8%) | 25 (96.2%) |
| Oman | 6 (12.0%) | 44 (88.0%) | 11 (12.1%) | 80 (87.9%) |
| Pakistan | 62 (27.8%) | 161 (72.2%) | 181 (21.5%) | 661 (78.5%) |
| Puerto Rico | 1 (2.9%) | 34 (97.1%) | 3 (12.5%) | 21 (87.5%) |
| Qatar | 16 (10.9%) | 131 (89.1%) | 23 (9.2%) | 228 (90.8%) |
| Saudi Arabia | 103 (15.3%) | 572 (84.7%) | 282 (16.7%) | 1402 (83.3%) |
| Sudan | 0 (0.0%) | 5 (100.0%) | 1 (14.3%) | 6 (85.7%) |
| Senegal | 4 (66.7%) | 2 (33.3%) | 5 (41.7%) | 7 (58.3%) |
| Trinidad & Tobago | 0 (0.0%) | 13 (100.0%) | 1 (11.1%) | 8 (88.9%) |
| Tunisia | 6 (15.0%) | 34 (85.0%) | 8 (10.3%) | 70 (89.7%) |
| Vietnam | 9 (12.2%) | 65 (87.8%) | 20 (10.0%) | 181 (90.0%) |
