# Supplementary material for: Linking citation and retraction data reveals the demographics of scientific retractions among highly cited authors
Source: PLoS Biol. 2025 Jan 30;23(1):e3002999. doi: 10.1371/journal.pbio.3002999 (PMC11781634; doi:10.1371/journal.pbio.3002999)
Supplement: Attachment — Submitted filename: repliesretra.docx [file pbio.3002999.s005.docx]

EDITORS’ COMMENTS:

You'll see that reviewer #1 says that the study is new and important, and simply has a few questions about methodology and a suggestions for some helpful diagrams. Reviewer #2 is also positive, but wants you to discuss more of the prior literature on retractions, to better justify a claim, and to point out that some retractions may not be down to the authors themselves. Reviewer #3 wants you to formulate clearer research questions, questions the point of discussing countries with very low publication rates, questions the rationale behind looking at citations in such a recent year as 2023, and wants more detail about name disambiguation. Reviewer #4 says that the paper is important, but thinks that it needs re-framing and clearer motivation (is it about the database, or is it about the retractions?), and wants more clarity on where the responsibility for retraction lies.

IMPORTANT: My diagnosis here is that the concerns raised by reviewer #3 and #4 are a natural consequence of my previous request that you do a "quick and dirty" cosmetic re-framing of the paper before review, as we were much more interested in the retraction analysis than in the database per se, and wanted the reviewers to focus on that aspect. The reviewers seem to detect the resulting disconnect, so I see revision as an opportunity for you to complete the process of re-framing around the retraction aspect (e.g. by including clear research questions, as the reviewers suggest). Obviously the other concerns raised by the reviewers should also be addressed (and for clarity, we're still interested in the updated database, but the retraction analysis should take centre stage).

Reply: These are all excellent suggestions and we have followed the guidance of the reviewers to adapt the manuscript accordingly. Research hypotheses and questions are stated more clearly upfront in the introduction, the introduction and the discussion sections have been strengthened, and several relevant references have been added to place the work in better context. The focus has shifted fully to the retractions aspect, as we had discussed also in the previous communication.

REVIEWERS' COMMENTS:

Reviewer #1:

[identifies himself as David B Resnik]

This article develops databases that links retraction and citation data. The database can be useful for future research on retractions. The study is well designed and executed. The information provided is new and important. I have just a few questions/suggestions.

Reply: Thank you for the kind appreciation of our work.

1. How was the linkage achieved? Was this automated? Done manually by members of the research team reviewing articles?

Reply: As we clarify the linkage was automated based on reasons of retraction and shared doi information. We clarify that “This linkage process was automated and it resulted in a total of 39,468 matched records” and “This first filtering was automated and resulted in a set of 47,964 entries.”

2. Is this database publicly available? Where? Searchable?

Reply: Yes, the database is publicly available at the provided link and it is a searchable Excel file that can be used for any type of purposes. We clarify that “The full datasets are available at https://doi.org/10.17632/btchxktzyw.7”

3. You might want to include some diagrams as visual aids to the reader, such as the steps in your search and linkage of records.

Reply: We have added a diagram to make more clear-cut the steps that we followed.

Reviewer #2:

In their study, Ioannidis et al. conducted a bibliometric analysis of retractions among highly-cited authors, highlighting a surprising ratio of highly-cited scientists having at least one retraction. This study is generally interesting and holds some value. However, the work does suffer from some issues.

Firstly, the article primarily focuses on the retractions of highly-cited authors, but the background is too brief to provide a comprehensive understanding of the retraction landscape. The authors should consider reviewing the latest relevant articles for a broader background, such as the following literatures, https://www.nature.com/articles/d41586-023-03974-8, 10.1007/s11192-024-04992-7, 10.2478/jdis-2024-0012, 10.1016/j.xinn.2024.100593, 10.1016/j.heliyon.2024.e38620, as well as more literatures. This could enhance the significance of their work.

Reply: thank you for these excellent suggestions. We are now citing these papers and we have expanded accordingly the Introduction and discussion sections to present them and improve the context placement of our work. We have added specifically in the Introduction: “The number of retracted papers per year is increasing, with more than 10,000 papers retracted in 2023 (ref van Noorden Nature 2024). The countries with the highest retraction rates (per 10,000 papers) are Saudi Arabia (30.6), Pakistan (28.1), Russia (24.9), China (23.5), Egypt (18.8), Malaysia (17.2), Iran (16.7), and India (15.2) (same ref). However, retractions abound also in highly developed countries such as European countries (10.1007/s11192-024-04992-7). There has also been a gradual change in the reasons for retractions over time (10.2478/jdis-2024-0012): the classic, traditional types of research misconduct (falsification, fabrication, plagiarism, and duplication) that involved usually one or a few papers at a time have been replaced in the top reasons by large-scale, orchestrated fraudulent practices (paper mills, fake peer-review, artificial intelligence generated content). Clinical and life sciences account for about half of the retractions that are apparently due to misconduct ([Science map of academic misconduct](https://www.cell.com/the-innovation/pdf/S2666-6758(24)00031-6.pdf)), but electrical engineering/electronics/computer science (EEECS) have an even higher proportion of retractions per 10,000 published papers ([Science map of academic misconduct](https://www.cell.com/the-innovation/pdf/S2666-6758(24)00031-6.pdf)). Clinical and life sciences disciplines have the highest rates of retractions due to traditional reasons of misconduct, while EEECS disciplines have a preponderance of large-scale orchestrated fraudulent practices.” In the Discussion, we have expanded two previous paragraphs to add more references and to discuss findings in context. Specifically, these paragraphs now run as follows: “More than half of the top-cited scientists with retractions were in medicine and life sciences. However, high rates were seen also in several other fields. A previous mapping of retractions due to misconduct (ref) had found the highest rates of retracted papers in EEECS at 18 per 10,000, double the rate for life sciences. The EEECS scientific area corresponds in our mapping to scientific domains where we also found high concentrations of top-cited scientists with retracted papers, although the rates were lower than the rate in Clinical Medicine and in Biomedical Research. It is possible that medical and life science retractions are more likely to involve top-cited authors, while EEECS retracted papers have mostly authors who do not manage to reach top-cited status. EEECS retractions have a large share of artificial intelligence generated content and fake review (ref). Therefore, it is likely that such fraudsters aim for more modest citation records, or they are revealed before they reach highly cited status, although exceptions do exist (ref). Many scientific fields have minimal or no track records of retractions and some subfields such as alternative medicine, cancer research, and pharmacology exhibit rates of retractions double the rates exhibited by the life sciences overall. These differences might reflect the increased scrutiny and better detection of misconduct and major errors in fields that have consequences for health; differences in the intensity and types of post-publication review practices (ref); and the fact that quantifiable data and images in the life sciences are easier to assess for errors and fraud than many constructs in social sciences.” “Many developing countries have extremely high rates of top-cited authors with retracted papers. This may reflect problematic research environments and incentives in these countries, several of which are also rapidly growing their overall productivity (refs). The countries where we detected the highest rates of top-cited authors with retractions largely overlap with the countries that have also the highest number of retracted papers per 10,000 publications according to a previous mapping of retractions due to misconduct(ref). In fact, some of these countries such as India, China, Pakistan and Iran also have a large share of implausibly hyperprolific authors (ref). It would be interesting to see if removing some of the productivity incentives may reduce the magnitude of the problem in these countries.”

Secondly, the authors claimed that retractions are more prevalent in the life sciences compared to other fields. The authors may not reach this conclusion based solely on the proportion of top-cited scientists with retracted publications. This requires literature support or a comparison of retraction rates.

Reply: Our database is focused on the roughly one quarter of a million highly-cited scientists, but other studies of retractions at the level of literature/published papers also show a relative preponderance of retractions in the life sciences compared with other scientific fields, but also high retractions in EEECS. See the previous point’s response on how we have elaborated on this. References have been added appropriately.

Thirdly, the criteria used to filter journal errors may be inadequate. For instance, instances of partial fake peer review may not necessarily be linked to the authors. It could be due to factors like editors unexpectedly inviting a fake referee or encountering review mills (see 10.1007/s11192-024-05125-w). Additionally, some reasons for retractions, such as Falsification/Fabrication of Data, Contamination of Cell Lines/Tissues, Contamination of Materials, Duplication of Text, among others, were not explicitly categorized as author errors.

Reply: We clarify that “We tried to separate retractions where there was no author responsibility, but some retraction notes are vague and the separation may not be perfect. Even for types of reasons that seem to be author-related, exceptions may exist, e.g. in partial fake review, it could be that the editors unexpectedly invite a fake referee or encounter review mills (added reference).” Falsification/Fabrication of Data, Contamination of Cell Lines/Tissues, Contamination of Materials, duplication of Text are eligible categories and these retractions are included in our database.

Lastly, there are issues with the citation style in the references. For example, "Oransky I. Volunteer watchdogs pushed a small country up the rankings. Science (1979). 2018;362(6413):395" requires correction. Please review the references for accuracy.

Reply: thank you for noticing this. We have corrected this reference and checked the other references.

Reviewer #3:

This manuscript describes an update of a dataset of highly cited authors. The update also includes the addition of the number of retracted papers and their number of citations by the highly cited authors. I'm missing the research question of this manuscript. It might help to formulate clear research questions, e.g., how high is the percentage of highly cited authors with retracted papers, or do the retracted papers make some authors highly cited? I don't see the connection to biology. Maybe, the authors can include a research question that is related to biology. More specific comments follow below.

Reply: Thank you for your kind appreciation of our work. We think the paper is more appropriate for PLoS Biology given the fact that PLoS Biology is highly multidisciplinary and has a special interest focus on meta-research, covering all scientific fields in that regard (https://plos.org/plos-biology-meta-research/). We fully agree that it is important to formulate clear research questions and aims, therefore we have added in the Introduction the following so as to focus the paper more on the retractions rather than the updating of a database (as suggested also by the editors): “Here, we aimed to analyze the presence of any retracted papers due to reasons that are probably related to the authors of the retracted paper for all the top-cited scientists across all 174 subfields of science. Typical impact metrics for scientists revolve around publications and their citations. However, citation metrics need to be used with caution (6) to avoid obtaining over-simplified and even grossly misleading views of scientific excellence and impact. We therefore updated and extended databases of standardized citation metrics across all scientists and scientific disciplines (refs) to include information on retractions for each scientist. Systematic indicators of research quality and integrity are important to examine side-by-side with traditional citation impact data (refs). A widely visible list of highly-cited scientists issued annually by Clarivate based on Web of Science no longer includes any scientists with retracted publications (ref). In our databases, which cover a much larger number of scientists with more detailed data on each, we have added information on the number of retracted publications, if any, for all listed scientists. Given the variability of the reasons behind retraction, this information can then be interpreted by any assessors on a case-by-case basis with in-depth assessment of reasons, and circumstances of each retraction. Using our expanded databases, we aimed to answer the following questions: How commonly have top-cited scientists retracted papers? Are there any features that differentiate top-cited scientists with versus without retracted papers? Are specific scientific fields and subfields more likely to have top-cited scientists with retracted papers? Do some countries have higher rates of retractions among their top-cited scientists? Finally, how much do citations to and from retracted papers contribute to the overall citation profile of top-cited scientists? As we present these analyses, we also hope that this new resource will be useful for further meta-research studies that may be conducted by investigators on diverse samples of scientists and scientific fields.”

The authors state in the abstract without providing any reason: "It would be useful to generate databases where the presence of retractions can be linked to impact metrics of each scientist." They continue: "We have thus incorporated retraction data in an updated a Scopus-based database of highly-cited scientists (top-2% in each scientific subfield according to a composite citation indicator)." This is the other way around compared to the preceding sentence.

Reply: Thank you for noticing this inconsistency. As stated in our previous response, this section has been extensively revised.

Also, in the abstract, the authors state: "In several developing countries, very high proportions of top-cited scientists had retractions (highest in Senegal (66.7%), Ecuador (28.6%) and Pakistan (27.8%) in career-long citation impact lists). Variability in retraction rates across fields and countries suggests differences in research practices, scrutiny, and ease of retraction." Especially, in the case of Senegal and Ecuador, this is statistics on very small numbers.

Reply: This is true and we comment on this in the text.

I do not see the benefit of analyzing the single most recent year (i.e., 2023) as this year's publications have had far too few time to be cited and generate impact.

Reply: Thank you for your observation. To clarify, the data for the most recent year (2023) refer to citations received in 2023 by papers published in any prior year, not solely to papers published in 2023. This approach is clearly outlined and supported by the background references, which detail the methodology used in constructing the citation databases.

The h/hm index has been mentioned (e.g., on page 6) but was not explained.

Reply: As we state, a higher ratio of h/hm index indicates higher co-authorship levels. We have added a footnote also in the relevant Table, where we explain: “The Schreiber hm index is constructed in the same way as the Hirsch h-index but considers also co-authorship. The more extensive the co-authorship, the more the hm index will deviate from (and become smaller than) the h-index.”

On page 9, the authors state: "Many developing countries have extremely high rates of top-cited authors with retracted papers. This may reflect problematic research environments and incentives in these countries, several of which are also rapidly growing their overall productivity (refs). In fact, some of these countries such as India, China, Pakistan and Iran also have a large share of implausibly hyperprolific authors (ref). It would be interesting to see if removing some of the productivity incentives may reduce the magnitude of the problem in these countries." I wonder if part of the "implausibly hyperprolific authors" and "extremely high rates of top-cited authors with retracted papers" might be due to a problem of the author name disambiguation. Also, no details regarding the author name disambiguation method were provided, like for the indicators used for ranking scholars.

Reply: We have added in the Discussion: “As discussed previously, Scopus has high precision and recall (23), but some errors do exist in author ID files. In the past, some Asian author IDs have very high numbers of papers because more than one author were merged in the same ID file. However, this is no longer the case and Asian name disambiguation in Scopus is currently as good or even better than European/American names (ref).”

Reviewer #4:

[identifies himself as Sean C. Rife]

The submitted manuscript investigates the increasing prevalence of retractions in scientific literature, which, despite their growth, still represent a small fraction of published works. By linking retraction data from the Retraction Watch database with citation metrics from Scopus, the authors found that a notable percentage of highly cited scientists had at least one retraction, with notable variations across disciplines and countries. The authors note that retractions are more frequent in the life sciences and highlight the necessity for careful interpretation of retraction data, as they do not always indicate misconduct, thereby providing a valuable resource for understanding scientific practices and enhancing research evaluation. I think this is an important paper that warrants publication. However, it could be improved in a number of ways, which I outline below. I also wonder if this paper might be better suited for PLOS ONE (although it would not qualify in my mind as suitable with only minor revisions, hence my lack of response to the earlier question of whether it would be "suitable for another PLOS journal with only minor revisions"), given its application to a wide array of scientific fields.

Reply: Thank you for your kind appreciation of our work. We think the paper is more appropriate for PLoS Biology given the fact that PLoS Biology is highly multidisciplinary and has a special interest focus on meta-research, covering all scientific fields in that regard.

Broadly, my concern is that I didn't get a clear understanding of the purpose of the paper. Is it supposed to elucidate the extent to which highly-cited authors have their papers retracted (and associated variables such as field, region, etc.), describe a newly-published dataset, or both? At present it reads like it is straddling the line between the two, which makes it somewhat difficult to follow. This could, perhaps, be improved by adding a simple statement outlining the purposes of the paper explicitly, early on, but I think the paper would benefit from a more thorough revision that makes the purpose clear at every stage.

Reply: Thank you for this excellent suggestion. As stated also by the editors and by reviewer 3, this is indeed highly desirable. The Introduction has been extensively rewritten and expanded. The last paragraph now ends as follows: “Using our expanded databases, we aimed to answer the following questions: How commonly have top-cited scientists retracted papers? Are there any features that differentiate top-cited scientists with versus without retracted papers? Are specific scientific fields and subfields more likely to have top-cited scientists with retracted papers? Do some countries have higher rates of retractions among their top-cited scientists? Finally, how much do citations to and from retracted papers contribute to the overall citation profile of top-cited scientists? As we present these analyses, we also hope that this new resource will be useful for further meta-research studies that may be conducted by investigators on diverse samples of scientists and scientific fields.”

I was also somewhat confused by the focus in various places on responsibility on the part of authors. The Method section might benefit from a brief explication of the authors' intentions with the filtering they applied. I presume the goal is to limit the analyses to instances in which the author(s) in question are responsible for errors or malfeasance, but then on p. 10 the authors state that they "make no judgment calls in our databases on the ethical nature of the retractions"; but then, do they not - at least implicitly - do so in the paper? Or am I assuming too much? This is also complicated (as the authors note in multiple instances) by the fact that many of the authors they identify may not be responsible for the elements of the papers that justified their retraction.

Reply: Thank you for discussing this important point. We try to exclude retractions where the journal or publisher clearly made an error. When it comes to the eligible retractions, the authors may have committed misconduct or they may have made honest errors. Moreover, in a paper it could be that only one or a few authors committed misconduct and the others were not aware of it. Or even for honest errors, the same may apply: only one or a few authors may be primarily responsible. We have clarified that “make no judgment calls in our databases on the ethical nature of the retractions, e.g. whether they represent misconduct or honest error by the authors”.

A few minor points:

- The authors note on p. 9 that retracted works often continue to be cited after they have been retracted. This is certainly problematic to the extent that the citing authors are unaware of the retraction, but there are also valid reasons to knowingly cite a retracted work (e.g., to discuss the nature/implications/etc. of the retraction).

Reply: We have clarified “and these citations are typically such that they suggest that the citing authors are unaware of the retraction rather than citing the paper to comment on its retraction.”

- The authors discuss paper mills in a number of places. A definition would be helpful.

Reply: We have added a definition: “massive production of papers that are typically fake/fabricated and where authors may buy authorship in their masthead”.

- The authors mention that some authors may be able to "game the system" re: publishing. An example would be helpful.

Reply: An example has been added: “e.g. have honorary authorship as department chair”.

- The authors note that retractions are more common in the life sciences and note that this might be due to increased scrutiny in these fields. This should probably be stated as a higher percentage, as a simple higher rate could be due to base rates (this is reflected elsewhere in the manuscript - just thinking it should be stated as a higher percentage here).

Reply: agreed, this has been clarified, see also our extensive response to a similar comment by reviewer 2.
