# Supplementary material for: Linking citation and retraction data reveals the demographics of scientific retractions among highly cited authors
Source: PLoS Biol. 2025 Jan 30;23(1):e3002999. doi: 10.1371/journal.pbio.3002999 (PMC11781634; doi:10.1371/journal.pbio.3002999)
Supplement: Attachment — Submitted filename: responsecomments.docx [file pbio.3002999.s006.docx]

a) Please change your Title to something more explicit, including an active verb. We suggest the following: "Linking citation and retraction data reveals the demographics of scientific retractions among highly-cited authors"

Reply: We have changed the title, as suggested.

b) You say that you received no specific funding for this work. Can you please confirm that this is indeed the case?

Reply: Yes, no funding was received specifically for this work.

c) Please address my Data Policy requests below; specifically, we need you to supply the numerical values underlying Figs 2AB, as a supplementary data file.

Reply: We are providing the numerical values underlying Figures 2A and B as a supplementary data file.

d) Please cite the location of the data clearly in the legend to Figure 2, e.g. “The data underlying this Figure can be found in S1 Data.”

Reply: done.

e) The Academic Editor wants you to include some RRIDs to improve long-term "findability" of the information. Specifically, they suggest the following instances: "To add the new information on retractions, we depended on the most reliable database of retractions available to date, the Retraction Watch database (RWDB, RRID:SCR_000654) which is also publicly freely available through CrossRef (RRID:SCR_003217)." and "Following this filtering process, we linked the retraction records to Scopus (RRID:SCR_022559) using the digital object identifier (DOI) of the original paper..."

Reply: done, as suggested.

f) Where you say "...publications (p<0.001 by Mann-Whitney U..." please report:

• which tool you ran your stats with (and the RRID and version of the tool) • U-statistic (or z-statistics for large groups), exact p-values, sample and group sizes • effect size • descriptive statistics

Reply: We have clarified this: “in R version 4.4.0 (RRID: SCR_001905)”. We can provide the log of running the tests, if requested, showing the W from which the U is derived, but such detail would be confusing for the general reader.

g) There's a typo in some of the new text ("Carrer-long impact counts" instead of "Career-long impact counts").

Reply: corrected.

h) Please make any custom code available, either as a supplementary file or as part of a DOI'd data deposition (e.g. in Zenodo). For example, I see that you describe the linkage of RetractionWatch entries to Scopus as being automated, so there is presumably a pipeline that performed this linkage? It would also be helpful if a more detailed description of how this linkage was performed were included in the manuscript itself.

Reply: We have added the following detailed description: “We tagged articles as retracted by linking retraction records to their corresponding entries in Scopus (RRID:SCR_022559). Initially, this linking is achieved by matching the OriginalPaperDOI with a DOI in Scopus. For retracted articles that do not have a direct DOI match, we employ an alternative strategy using the title and publication year, allowing for a one-year discrepancy due to variations in the recorded publication year. To enhance the accuracy of our linking process, we perform data sanitization on both databases. DOIs are standardized by removing redundant prefixes and extraneous characters. Titles are normalized by stripping all non-alphanumeric characters and converting them to lowercase. Additionally, to avoid erroneous matches, especially with shorter titles, we impose a minimum length requirement of 32 characters for title matching. The code that demonstrates the linking strategy is published along the dataset at https://elsevier.digitalcommonsdata.com/datasets/btchxktzyw/7.”

As you address these items, please take this last chance to review your reference list to ensure that it is complete and correct. If you have cited papers that have been retracted, please include the rationale for doing so in the manuscript text, or remove these references and replace them with relevant current references. Any changes to the reference list should be mentioned in the cover letter that accompanies your revised manuscript.

Reply: checked and some typos were corrected (highlighted), references are still the same.
